# Potential Diagnostic Value of Serum p53 Antibody for Detecting Esophageal Cancer: A Meta-Analysis

**DOI:** 10.1371/journal.pone.0052896

**Published:** 2012-12-28

**Authors:** Jun Zhang, Zhiwei Xv, Xuefeng Wu, Ke Li

**Affiliations:** Department of Preventive Medicine, Shantou University Medical College, Shantou, Guangdong, China; Tehran University of Medical Sciences, Islamic Republic Of Iran

## Abstract

**Background:**

Mutant p53 protein overexpression has been reported to induce serum antibodies against p53. Various studies assessing the diagnostic value of serum p53 antibody in patients with esophageal cancer remain controversial. This study aims to comprehensively and quantitatively summarize the potential diagnostic value of serum p53 antibody in esophageal cancer.

**Methods:**

We systematically searched PubMed and Embase until 31st May 2012, without language restriction. Studies were assessed for quality using QUADAS (quality assessment of studies of diagnostic accuracy). Positive likelihood ratio (PLR) and negative likelihood ratio (NLR) were pooled separately and compared with overall accuracy measures diagnostic odds ratio (DOR) and symmetric summary receiver operating characteristic (sROC). The PLR and NLR and their 95% confidence interval (CI) were calculated using a fixed effects model according to the Mantel-Haensed method and random effects model based on the work of Der Simonian and laird, respectively.

**Results:**

Fifteen studies (cases = 1079, controls = 2260) met the inclusion criteria for the meta-analysis. Approximately 53.33% (8/15) of the included studies were of high quality (QUADAS score≥8), which were retrospective case-control studies. The summary estimates for quantitative analysis of serum p53 antibody in the diagnosis of esophageal cancer were PLR 6.95 (95% CI: 4.77–9.51), NLR 0.75 (95%CI: 0.72–0.78) and DOR 9.65 (95%CI: 7.04–13.22). However, we found significant heterogeneity between NLRs.

**Conclusions:**

The current evidence suggests serum p53 antibody has a potential diagnostic value for esophageal cancer. However, its discrimination power is not perfect because of low sensitivity.

**Impact:**

These results suggest that s-p53-antibody may be useful for monitoring residual tumor cells and for aiding in the selection of candidates for less invasive treatment procedures because of the high specificity of s-p53-antibody. Further studies may need to identify patterns of multiple biomarkers to further increase the power of EC detection.

## Introduction

Esophageal cancer, composed of squamous cell carcinoma and adenocarcinoma, is the eighth most common cancer worldwide, constitutes 6.13% of all digestive system cancer, with 482,300 new cases annually, and has the sixth highest cancer mortality, with 406,800 deaths registered in 2008 worldwide [1]. Furthermore, 17,460 cases of esophageal cancer are expected to be newly diagnosed in 2012, with 15,070 estimated deaths accounting for 86% of all estimated new cases [2]. During the early stages of the esophageal cancers, patients are usually asymptomatic and go undetected until they are incurable. The prognosis of this disease is unfavorable in spite of advances in therapies. However, if patients are diagnosed at an early stage, the overall survival could be significantly improved, with a 5-year survival rate of up to 90% [3]. Although current diagnostic procedures (pathologic examinations of resected specimens) improve the accuracy of the diagnosis, such procedures are often invasive, unpleasant, inconvenient and expensive. Hence, there is a great need for identification of novel non-invasive diagnostic methods for early tumor detection.

Mutations in the tumor suppressor gene p53 are the most commonly observed genetic abnormalities in human cancers [4]. The protein product of the p53 gene is a nuclear phosphoprotein expressed in normal cells. In the serum of healthy subjects the presence of p53 protein and anti-p53 antibodies are extremely rare [5]. Mutations in this gene cause an accumulation of non-functional proteins, due to increased stability and a longer half-life of several hours compared with 20 min for the wild-type p53, which can be detected by immunoassay [5]. The accumulated protein then acts as an antigen, with subsequent development of antibodies (anti-p53 antibodies), which are detectable in tissues, sloughed cells, blood, and other body ﬂuids [5]. With the development of molecular biotechnology, a large number of studies on the potential diagnostic value of serum p53 antibody for esophageal cancer have been published and have reported varying results.

In order to elucidate whether serum p53 antibody can be used as a serological marker in the diagnosis of esophageal cancer. In this study, we conducted a systematic review and meta-analysis to appraise the accuracy of serum p53 antibody for esophageal cancer screening.

## Materials and Methods

### Search Strategy and Study Selection

We searched PubMed and EMBASE to identify suitable studies prior to 31st May, 2012. No start data limit was applied. The search term was ‘esophageal neoplasm’, ‘blood OR serum’, ‘seropositive OR serum antibody’, p53 or TP53’ (please see Table S1), without language restriction. Articles were also identified by use of the related articles function in PubMed and the references of identified articles were searched manually.

Two reviewers (J Zhang and ZW Xv) independently inspected the title and abstract of each citation to identify those studies that were likely to report the diagnostic value of serum p53 (s-p53) antibody and then obtained the full text. Disagreements about study selection were resolved by consensus. The full text was retrieved for articles that could not be excluded based on title and abstract to determine inclusion. Inclusion criteria for the primary studies were as follows: (i) participants: all cases must have been diagnosed by a gold standard (pathologic examinations of biopsied specimens), serum must have been collected for anti-p53 analysis before any treatment, e.g. chemotherapy or radiotherapy, and controls were without other cancers, (ii) index test: studies evaluated the diagnostic value of s-p53 antibody in esophageal cancer, (iii) outcome: studies reported the positive values of the cases and controls, and the results of an individual study on diagnostic accuracy can be summarized in a 2×2 table, (iv) study design: No restrictions were made with respect to study design (cross sectional, case control, corhort study) or data collection (prospective or retrospective).

### Assessment of Methodological Quality

Two dependent reviewers (J Zhang and ZW Xv) used 11 items of published QUADAS (quality assessment for studies of diagnostic accuracy ) guidelines as a tool to assess the included studies, and disagreements were resolved by consensus. The 11 items were recommended by the Cochrane Collaboration Methods group on screening and diagnostic tests [6]. The items got a “1” score if the item score was “yes”, and aggregate scores were 11. Items included covered patient spectrum, reference standard, disease progression bias, verification bias, review bias, clinical review bias, incorporation bias, test execution, study withdrawals, and indeterminate results. The QUADAS tool is presented together with guidelines for scoring each of the items included in the tool.

### Data Extraction and Management

The final eligible articles were reviewed independently by two reviewers (J Zhang and ZW Xv), and disagreements were resolved by consensus. The following characteristics studies were extracted: (i) basic information: systematic review date, conductor, study ID and study details (first author, year of publication, country of publication), (ii) study eligibility: based on inclusion/exclusion criteria to assess again and to record the reason for the excluded studies, (iii) methods of the study characteristics: participants’ inclusion/exclusion criteria, ethnicity, disease stage, histology stage, diagnostic guidelines, type of control, (iv) index tests: the extraction time and storage temperature of the sample, assay method, cut-off value, blind, a detailed report of the assay procedure, (v) outcome: the positive value of the cases and controls, and other comparison data (e.g. mean age, sex ratio, smoking, drinking) between cases and controls. If data from any of the above categories were not reported in the primary article, items were treated as “not reported.” We did not contact the author for further details.

### Statistical Analyses

We used standard methods recommended for meta-analysis of diagnostic test evaluations [7]. The statistical analysis was based on the following steps [7]: 1-presentation of the results of individual studies. Reporting the main results of all included studies is an essential part of each review. Each study is presented with background information (year of publication, country, selection of the patients and methodological characteristics). 2-searching for the presence of heterogeneity. Most diagnostic reviews show considerable heterogeneity in the results of included studies. When different studies have largely different results, this may result from either random error or heterogeneity due to differences in clinical or methodological characteristics of studies. A chi-square test can be used to statistically test the presence of heterogeneity in study results. 3-testing of the presence of cut-off threshold effects. Estimates of diagnostic accuracy differ if not all studies use the same cut-off point for a positive test result or for the reference standard. Variation in the parameters of accuracy may be partly due to variation in cut-off point. We can test for the presence of a cut-off point effect between studies by calculating a Spearman correlation coefficient between sensitivity and specificity of all included studies. 4-dealing with heterogeneity. Subgroup analysis and meta regression could be conducted to detect the heterogeneity between studies. 5-statistical pooling: positive likelihood ratio (PLR), negative likelihood ratio (NLR) and their 95% confidence interval (CI) were calculated using a fixed effects model according to the Mantel-Haensed method and random effects model based on the work of Der Simonian and laird [8], respectively. The likelihood ratio incorporates both the sensitivity and specificity of the test, and provides a direct estimate of how much a test result will change the odds of having a disease [9]. The PLR indicates how much the odds of the disease increase when a test is positive [9], and the NLR indicates how much the odds of the disease decrease when a test is negative. Likelihood ratios of >10 or <0.1 generate large and often conclusive shifts from pretest to posttest probability (indicating high accuracy) [9]. According to Honest H, Khan KS [10], sensitivity and specificity are considered inappropriate for meta-analyses, as they do not behave independently when they are pooled from various primary studies to generate separate averages.The accuracy measure used was the diagnostic odds ratio (DOR) computed by the Moses’s constant of linear method, which indicates the change in diagnostic performance of the test under study per unit increase in the covariant [11]. The DOR is a single indicator of test accuracy that combines the data from sensitivity and specificity into a single number [12]. The value of DOR ranges from 0 to infinity, with higher values indicating better discriminatory test performance (higher accuracy) [12]. A DOR of 1.0 indicates that a test does not discriminate between patients with the disorder and those without it [12]. Summary receiver operating characteristic curves were used to summarize overall test performance, and the area under the SROC curve (AUC) was calculated. The SROC curve has been recommended to represent the performance of a diagnostic test, based on data from meta-analysis, and the area under the SROC curve (AUC) is not only useful to summarize the curve, but also quite robust to heterogeneity [13], [14]. A prior study [15] showed that to demonstrate excellent accuracy, the AUC should be in the region of 0.97 or above. An AUC of 0.93 to 0.96 is very good; 0.75 to 0.92 is good. An AUC less than 0.75 can still be reasonable, but the test has obvious deficiencies in its diagnostic accuracy. The potential problem associated with sensitivities and specificities of 100% are solved by adding 0.5 to all cells of the diagnostic 2×2 table [7].

We used a chi-squared test to detect statistically significant heterogeneity. Between-study heterogeneity was assessed using I^2^,according to the formula: I^2^ = 100%×(Cochran Q –degrees of freedom)/Cochran Q [16]. To detect cut-off threshold effects, the relationship between sensitivity and specificity was evaluated by using the Spearman correlation coefficient r. Possible sources of heterogeneity were investigated by meta regression, which used a generalization of Littenberg and Moses linear model weighted by the inverse of the variance [11]. Also, we conducted subgroup analysis. In order to evaluate the statistical outcome validity, we detected the pooled outcome by sensitivity analysis. Since publication bias is of concern for meta-analysis of diagnostic studies, we tested for the potential presence of this bias using funnel plots [17]. Publication bias is assessed visually by using a scatter plot of the inverse of the square root of the effective sample size (1/ESS1/2) versus the diagnostic log odds ratio (lnDOR) which should have a symmetrical funnel shape when publication bias is absent [18]. Formal testing for publication bias may be conducted by a regression of lnDOR against 1/ESS1/2, weighting by ESS [18], with P<0.05 for the slope coefficient indicating significant asymmetry. All analyses were undertaken using Meta DiSc statistical software (version 1.4; Ramon y Cajal Hospital, Madrid,Spain) [19] and stata SE12.0 software (Stata Corporation).

## Results

### Results of the Search and Characteristics of the Studies

**Figure 1:**
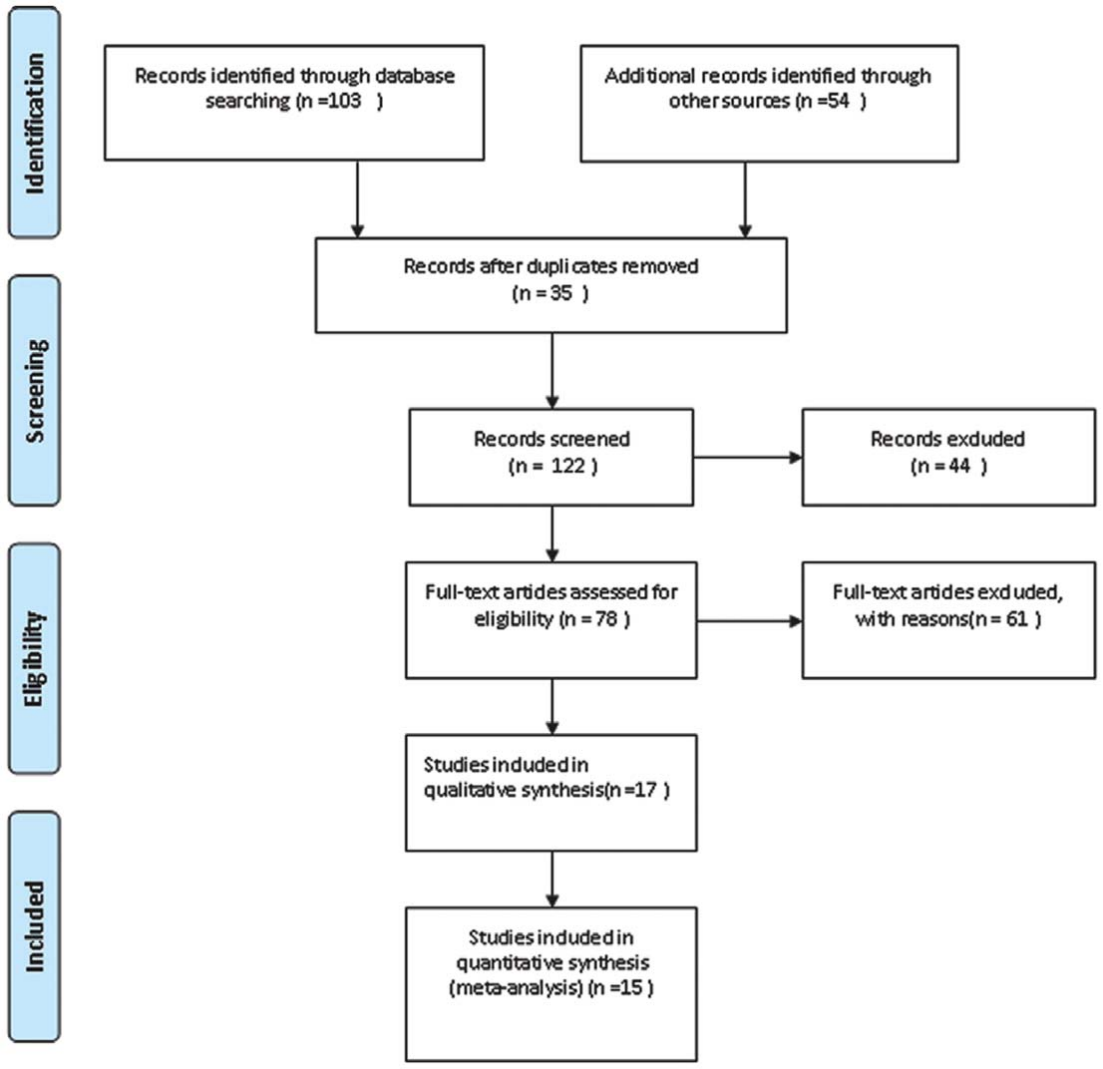
Flow chart of study selection by using electronic database and other sources.

Abstracts and titles of 103 primary studies were identified for initial review using the search strategies as described in Fig. 1. After reading the titles and abstracts, 25 unrelated articles were excluded, resulting in 78 full-texts on the role of s-p53 antibody in the diagnosis of EC being obtained, which were selected by inclusion and exclusion. Of these publications, 16 articles, including a review and case report, were excluded because they provided insufficient information. An additional 23 were excluded because there was no control, and 22 studies were excluded because they focused on the p53 gene and p53 protein and did not detect s-p53 antibody. As a consequence, only 17 publications were considered to be eligible for inclusion in the analysis, however, two studies [20], [21] with controls were subsequently excluded because they did not allow the calculation of sensitivity or specificity. Finally, the remaining 15 [5], [22]–[35] articles based on cases with EC and controls without EC were available for meta-analysis and the diagnostic characteristics of these studies, along with QUADAS scores, are outlined in Table 1 and Table 2. These studies followed several different characteristics. The studies included were conducted in different countries, five [24], [25], [31]–[33] of 15 studies were conducted in Japan, five [5], [22], [27], [28], [35] in China, two [30], [34] in India, one [29] in Germany, one [26] in Poland and one [23] study being from America. The publication years ranged from 1998 to 2010. Five studies [22], [26], [29], [31], [33] choose consecutive patients, one [5] choose random patients, and nine did not report related information. All of the 15 studies were retrospective, seven [22], [24]–[26], [30], [31], [35] provided the TNM stage and 6 [22], [25], [26], [30], [33], [35] provided the histology stage. Thirteen of the studies included health volunteers as a control, and the remaining two studies [23], [33] included health volunteers and patients with benign disease as controls.

**Table 1:** Main characteristics and results of the 15 eligible studies.

Author/Year	Country	Reference	Assay	Cut-off	TP*	FP*	FN*	TN*
		standard	method					
Helen M./1998 [23]	America	Histology	Immunoblot,	Unknown	15	1	54	18
			precipitation					
Parashar K./1998 [34]	India New Delhi	Unknown	ELISA*	Unknown	6	0	14	20
Shimada H./2000 [33]	Japan	Histology	ELISA*	Index> = 1.1,Abs*	14	0	21	88
				orption> = 1.6				
Hagiwara N./2000 [24]	Japan	Histology	Sandwich	Unknown	13	0	33	13
			ELISA*					
Ralhan R./2000 [30]	India Chandigarh	Histology	ELISA*	Unknown	36	4	24	46
Kozlowski M./2001 [26]	Poland	Unknown	ELISA*	Index> = 1.1	20	0	55	10
Shimada H./2002 [31]	Japan	Histology	ELISA*	1.3 U/ml	28	7	77	146
Shimada H. 2003 [32]	Japan	Unknown	ELISA*	1.3 U/ml	90	23	211	371
Wang M.H./2004 [35]	China	Histology	ELISA*	Index> = 1.1,Abs*	18	0	20	20
				orption> = 1.6				
Hiroyuki K./2005 [25]	Japan	Histology	ELISA*	1.3 U/ml	18	0	39	17
Megliorino R./2005 [28]	China	Histology	ELISA*	Normal	11	2	66	82
				mean+3SD				
Looi K./2006 [27]	China	Unknown	ELISA*	Normal	5	1	66	81
				mean+3SD				
Muller M./2006 [29]	Germany	Histology	Immunoblot	Unknown	10	0	40	436
Cai H.Y./2008 [22]	China	Histology	ELISA*	Unknown	18	0	28	30
Wu M./2010 [5]	China	Unknown	ELISA*	Unknown	4	9	25	870

Note: ELISA* = Enzyme-linked immunosorbent assay; TP* = true positives, FP* = false positives, FN* = false negatives, TN* = true negatives; Abs* = Antibody.

**Table 2:** Main characteristics of the 15 eligible studies.

Author/Year	Consecutive/random	Histology Well/Moderate/poorly/other	Sample collection time	Stage I(%)	QUADAS
Henlen M./1998	Unknown	Unknown	Unknown	Unknown	8
Parashar K./1998	Unknown	Unknown	Unknown	Unknown	6
Shimada H./2000	Consecutive	7/16/10/0	Before treatment	Unknown	10
Hagiwara N./2000	Unknown	Unknown	Before treatment	6/46 (13.0%)	7
Ralhan R./2000	Unknown	26/20/12/0	Before treatment	6/60 (10.0%)	9
Kozlowski M./2001	Consecutive	4/32/28/11	Before diagnosis	4/75 (5.3%)	7
Shimada H./2002	Consecutive	Unknown	Unknown	50/105 (47.6%)	8
Shimada H./2003	Unknown	Unknown	Unknown	Unknown	6
Wang M.H./2004	Unknown	12\12\14\0	Before treatment	10/38 (26.3%)	9
Hiroyuki K./2005	Unknown	13/19/17/8	unknown	13/57 (22.8%)	8
Megliorino R./2005	Unknown	Unknown	Before chemotherapy	Unknown	8
Looi K./2006	Unknown	Unknown	Before diagnosis	Unknown	7
Muller M./2006	Consecutive	Unknown	unknown	Unknown	7
Cai H.Y./2008	Consecutive	15/17/14/0	Before chemotherapy	10/46 (21.7%)	8
Wu M./2010	Random	Unknown	Unknown	Unknown	6

Note: QUADAS: quality assessment of studies of diagnostic accuracy.

### Methodological Quality of Included Studies

Quality assessment based on QUADAS guidelines was conducted on all 15 studies included for systematic review. Of the 15 eligible studies, eight [22], [23], [25], [28], [30], [31], [33], [35] had QUADAS score≥8, four [24], [26], [27], [29] had a QUADAS score = 7 and three [5], [32], [34] had a QUADAS score = 6. In total included studies (please see Figure S1), exceeding 50% had high quality on the acceptable reference standard and about 40% had high quality on the acceptable delay between tests. And about 60% and 80% had high quality in the items of incorporation avoided and uninterpretable results reported, respectively. In addition, five items (partial verification avoided, differential verification avoided, reference standard results blinded, relevant clinical information, withdrawals explained) had 100% high quality. However, exceeding 75% of the publications had low quality on the representative spectrum. And all of the 15 eligible studies showed the item of the index test results blinded unclear.

### Threshold Effect

Computation of the Spearman correction coefficient between the logit of sensitivity and logit of 1-specificity of s-p53 antibody was 0.125 (P = 0.667), indicating no threshold effect [36], and the positive correlation had no statistical significance.

### Diagnostic Accuracy

**Figure 2:**
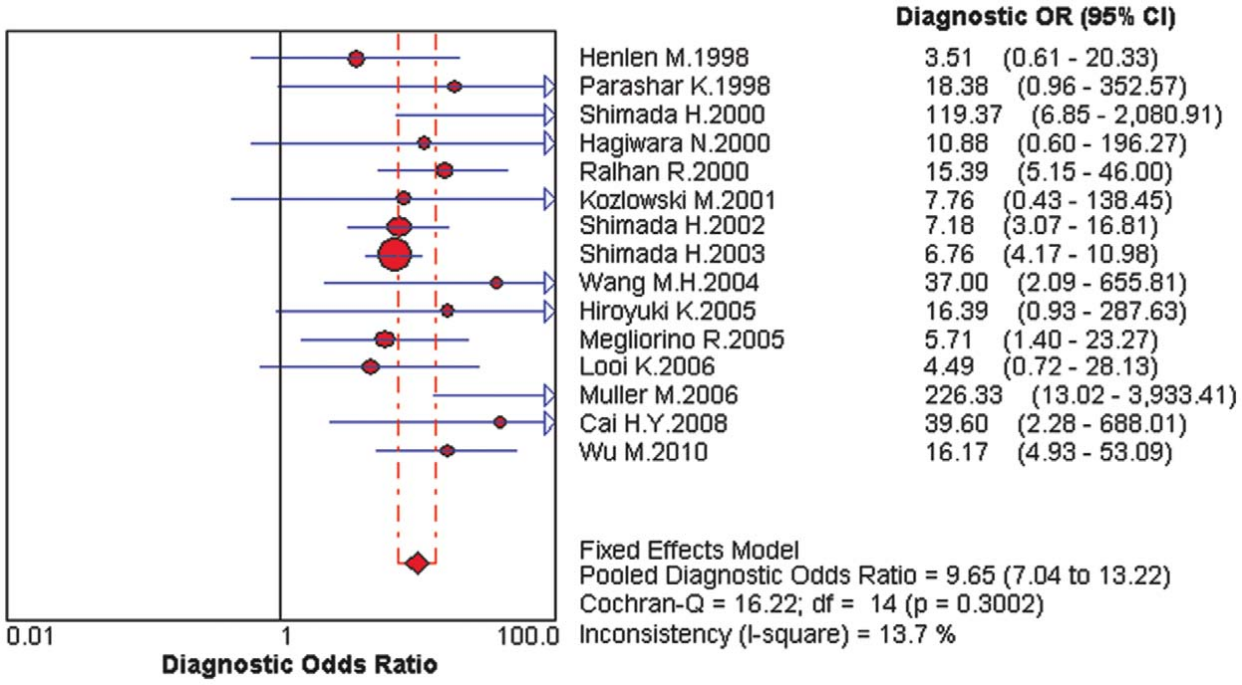
Forest plot of estimates of the diagnostic odds ratio (DOR) for s-p53 antibody in the diagnosis of EC. The point estimates of the diagnostic odds ratio from each study are shown as solid circles. Error bars are 95% confidence intervals.

**Figure 3:**
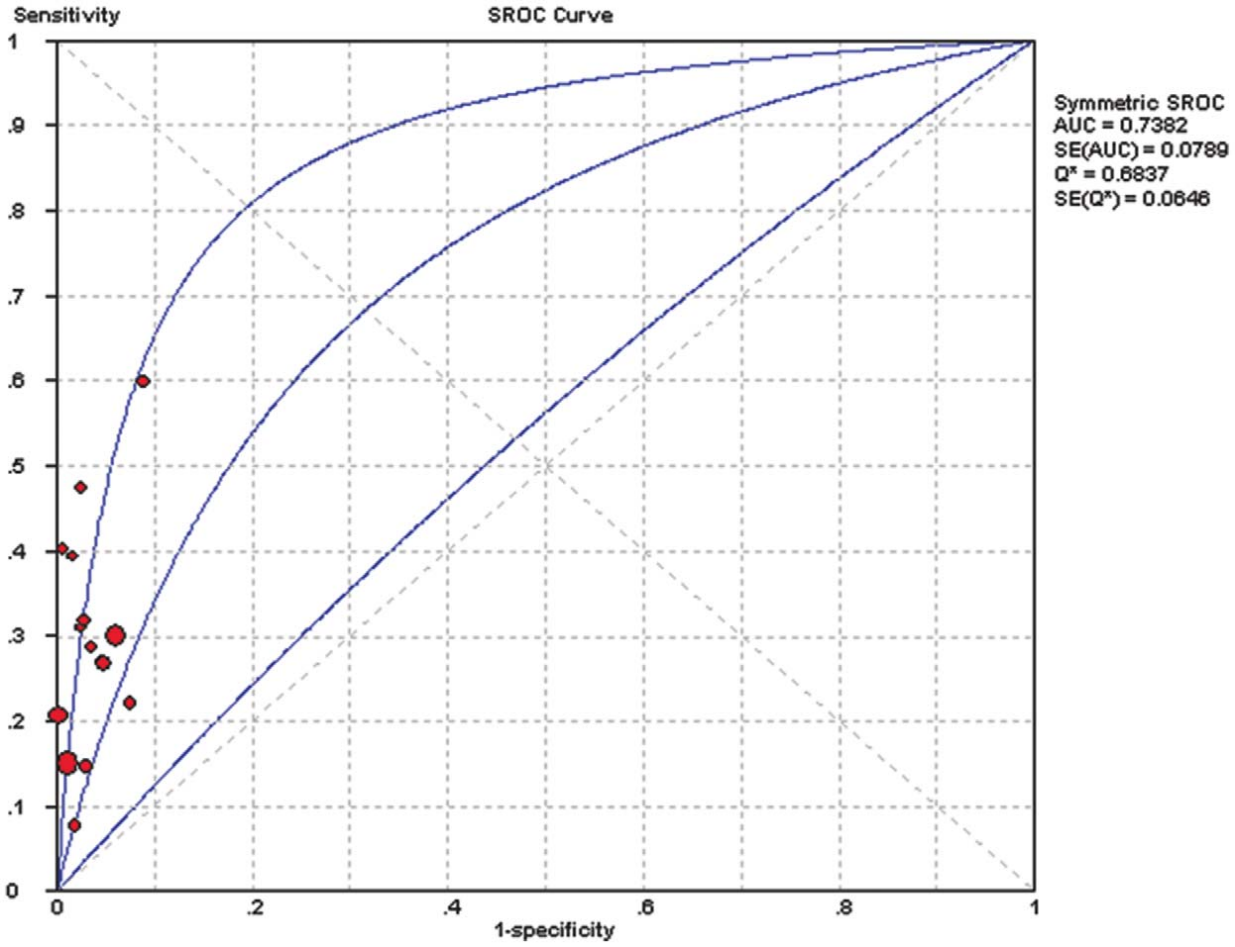
Summary receiver operating characteristic curves for s-p53 antibody in the diagnosis of EC. Each solid circle represents each study in the meta-analysis. The size of each is indicated by the size of the solid circle. The weighted (solid line) and unweighted (dashed line) regression summary receiver operating characteristic curves summarize the overall diagnostic accuracy.

For all studies, the pooled DOR was 9.75 (95%CI: 6.47–14.71), heterogeneity chi-squared = 16.22 (p = 0.300) and I^2^ = 13.70%. There did not appear to be any major qualitative evidence for heterogeneity between studies, as assessed by inspection of the forest plot (Fig. 2). The DOR value approximate to 10 indicated that the s-p53 antibody could be useful biomarker for EC patients diagnosis. Fig.3 presented the symmetrical SROC of s-p53 antibody, and the AUC was 0.74. In our study, the AUC of s-p53 antibody was 0.74, close to 0.75. Thus s-p53 antibody had reasonable accuracy in terms of differential diagnosis in cases of EC.

The range of the sensitivity and specificity were 15%–60% and 91%–100%, respectively (please see Figure S2). In the present study, a pooled PLR of 6.98 (95% CI: 5.18–9.34) suggests that patients with EC have a nearly 7-fold higher chance of being s-p53 antibody test-positive compared with patients without EC (please see Figure S3). Also, there were no heterogeneity between PLRs, heterogeneity chi-squared = 15.27 (p = 0.360) and I^2^ = 8.30%. Regarding NLR, we found significant heterogeneity for all of the eligible studies, heterogeneity chi-squared = 72.93 (p = 0.000) and I^2^ = 80.80%. The pooled negative likelihood ratio was 0.74 (95% CI: 0.68–0.81) (please see Figure S3).

### Possible Sources of Heterogeneity

The meta-regression and sub-group analyses were used to explore the overall heterogeneity and the possible sources of heterogeneity, which may include variation in method quality of the studies (QUADAS), assay method, the representation of the participants (stage I%), negative control, sample collection time among each study. Meta-regression indicated that above variables were not the sources of heterogeneity for s-p53-antibody (data not shown). The subgroup analysis results was show in Table 3, and the main source may be from assay method, the percentage of the stage I, negative control, sample collection time.

**Table 3:** Possible sources of heterogeneity of sub-group analysis.

Subgroup	(n)	PLR (95% CI)*	NLR (95% CI)*	DOR (95% CI)*
QUADAS	≥8(n = 8)	6.53 (4.05–10.05)	0.68 (0.58–0.79)	10.42 (5.61–19.34)
	<8 (n = 7)	8.40 (4.10–17.18)	0.80 (0.72–0.89)	10.08 (5.14–19.81)
Assay method	ELISA (n = 12)	6.71 (4.92–9.16)	0.73 (0.65–0.81)	9.37 (6.76–12.99)
	Other (n = 3)	9.94 (3.83–25.81)	0.80 (0.72–0.88)	12.71 (3.79–42.63)
Stage I%	>20% (n = 4)	8.63 (4.16–17.91)	0.68 (0.59–0.78)	11.89 (5.78–24.46)
	< = 20% (n = 3)	6.82 (2.96–15.72)	0.64 (0.43–0.94)	13.07 (4.89–34.89)
Negative control	Health (n = 13)	6.72 (4.95–9.14)	0.75 (0.68–0.82)	9.36 (6.77–12.95)
	Health +benign disease (n = 2)	10.51 (3.49–31.60)	0.72 (0.49–1.07)	13.53 (3.56–51.45)
Sample collection time	Before treatment (n = 6)	10.38 (5.44–19.81)	0.63 (0.49–0.81)	17.58 (8.65–35.73)
	Before diagnosis (n = 2)	5.26 (1.54–17.98)	0.86 (0.71–1.06)	6.20 (1.75–21.98)

Note:PLR (95% CI)* and DOR (95% CI)* was calculated using fixed effect model; NLR (95% CI)* was calculated using random effect model.

### Sensitivity Analysis and Publication Bias

**Figure 4:**
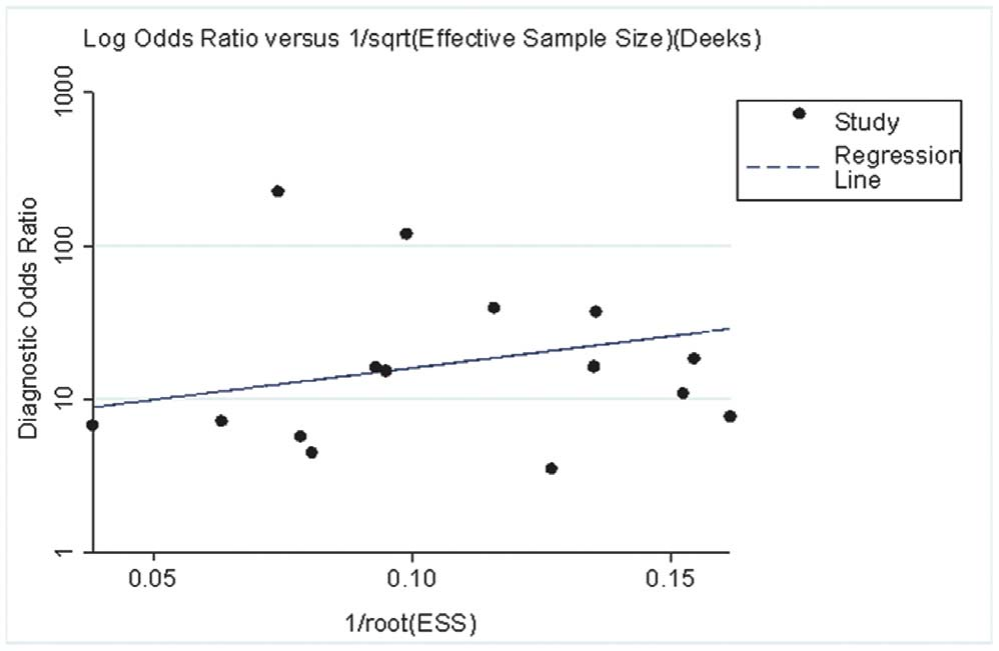
Funnel plot for the assessment of potential bias in s-p53 antibody assays. The funnel graph plots the DOR (diagnostic odds ratio) against the 1/root (effective sample size). The dotted line is regression line. The result of the test for publication bias was not significant (p = 0.305).

Sensitivity analysis was conducted in terms of statistical analysis methods, sample size, and study design. We used a random effect model to analysis the data again to replace the fixed effect model, however, the results produced no obvious changes. When we excluded the studies without matched cases and control sample size, the results were similar to the original results. In addition, we excluded the studies which studied various cancers that included EC and did not provide the detailed information of the participants, but this did not change the results. Although meta-analysis itself has some bias, the results showed no publication bias in this meta-analysis (p = 0.305). The funnel plots (Fig. 4) for publication bias also showed symmetry.

## Discussion

Our meta-analysis allow some conclusions based on available evidence: (1) patients with EC have higher chance of being s-p53 antibody test-positive compared with patients without EC; (2) the ratio of the odds of a positive test result among EC was approximate 10-folders to the odds of a positive test result among the non-EC. In brief, s-p53-antibody could be useful for the detection and diagnosis of EC, whereas it is imperfect.

As we all know, s-p53 antibody is not specific to EC. Positive correlations have been reported between p53 immunoreactivity and the presence of S-p53 Abs in patients with esophageal carcinoma [37], gastric carcinoma [38], colorectal carcinoma [39], and ovarian carcinoma [40]. A strong correlation was reported between p53 mutation and the presence of S-p53 Abs [37], [41]. Actually there a great deal of studies which have reported the presence of anti-p53 antibodies to be an indicator of diagnosis or poor prognosis in patients with bladder [42], [43], hepatocellular carcinoma [44]–[49], head and neck cancer [50]–[53], pancreatic [54]–[57]. The positive rates of S-p53 Abs were compatible to the rates of p53 mutation in those malignant tumors [58]. Studies of the molecular biology of malignant tumors have emphasized the importance of a number of protooncogenes and tumor suppressor genes in human malignancies. Thus, the search for biomarkers that can diagnose various types of malignancies is important for the better management of patients.

Early detection of EC is still a common problem in clinical practice. To our knowledge, there is no diagnostic biomarker for EC. Usually, histological examination is used to diagnose EC. More and more studies have been focused on the detection of s-p53 antibody in EC to evaluate the diagnostic and clinical usefulness of the anti-p53 antibody response as a serological marker. Several studies have reported that serum p53 antibodies (s-p53 Abs) are detected in different populations that are at increased risk for developing malignant disease [59]–[61]. S-p53 Abs can be used to follow the response of patients with malignant tumors during treatment [62]. Because the ELISA assay is a quick and convenient assay for detecting p53 genetic alterations, s-p53 Abs may serve as a useful marker for routine screening in EC patient groups. This is the first meta-analysis about s-p53 antibody and esophageal cancer screening. In the present study, 15 studies which including 1079 serum samples from EC patients and 2260 serum samples from controls without EC were eligible according to our inclusion criteria. Although all of the 15 eligible studies aimed to ensure the diagnostic accuracy of the s-p53 antibody, those studies could be only regarded as being in the early stage of diagnostic testing. In all 15 studies, EC patients diagnosed by histology were regarded as positive. However, the negative controls without EC who were healthy or had benign disease were not diagnosed by histology. In addition, the 15 studies did not report whether the investigators were blinded. Therefore, such non-strict designs could exaggerate the diagnostic accuracy and lead to bias due to unfavorable representation of the participants. Simultaneously, QUADAS, recommended by Cochrane, which can be used in systematic reviews of diagnostic accuracy studies, was used to evaluate the methodological quality of the included studies. Our meta-analysis showed that methodological quality of reports on diagnostic research of s-p53-antibody is moderate, as expressed by the QUADAS tool. Systematic reviewers are advised to use comprehensive searches to attempt to locate all relevant studies [63]–[65]. In our study, we did not find any publication bias (p = 0.305).

In meta-analysis, pooled indicators were usually used in the homogeneity study. In the present study, however, there were significant heterogeneity between NLRs, so it is not suitable to pool NLR (I^2^ = 80.8%). Therefore, the DOR and AUC were calculated for evaluating the potential diagnostic values of s-p53 antibody. DOR is difficult to be clinically interpreted, but useful from the statistical point of view in the assessment of the overall test accuracy in meta-analysis [66]–[68]. It is very important to note that the point estimates of PLR and DOR must evaluate carefully and the heterogeneity between NLRs should be searched and explained. As different cut-off values were used among the 15 included studies, we used the Spearman correlation coefficient to analyze the threshold effect. The result had no statistical significance (p = 0.66>0.05), indicating that a threshold effect was not the source of the heterogeneity. Nonetheless, the validation assay of s-p53 antibody used in each study was different; some used ELISA, others used immunoblot or both, adding additional heterogeneity. The spectrum of patients refers not only to the severity of the underlying target condition, but also to demographic features and to the presence of differential diagnosis and/or co-morbidity. It is therefore important that diagnostic test evaluations include an appropriate spectrum of patients for the test under investigation and also that a clear description is provided of the population actually included in the study [36]. The difference of the percentage of stage I patients between studies brought about spectrum bias and heterogeneity. Studies including healthy controls tend to show higher specificity than those recruiting patients with clinically suspected disease consecutively and prospectively in a representative clinical setting. Therefore, the distinct type of negative control may be a main sources of heterogeneity. The sample collection time varied widely among the studies. Four studies [24], [30], [33], [35] collected serum before treatment, seven studies [5], [23], [25], [29], [31], [32], [34] did not report, two studies [22], [28] collected serum before chemotherapy and two studies [26], [27] collected serum before diagnosis, respectively. The differentials of DOR between sample collection time subgroups indicated that different collection times also led to significant heterogeneity.

Although we tried to avoid the bias in the process of identifying studies, screening, assessing, data extraction, data analyses, etc; the present study has several limitations: First, we did not calculate the diagnostic accuracy for the early stage (stage I–II), in that sufficient raw data was not provided. Although we aimed to determine the screening power of the s-p53 antibody for the early diagnosis of the EC, EC patients regardless of disease stage were used to evaluate the diagnostic power because of the limitation of the information. There were also not available primary data to investigate the elevated or decreased s-p53 antibody values as a function of tumor type, histology, age, or degree. Second, all of the 15 included studies used healthy controls and only two studies (2/15) added benign disease, which strongly exaggerated the diagnostic accuracy. Actually, all of the 15 included studies lacked the appropriate matching of age, storage conditions, and location of obtaining and handling of the samples between case and control. It is significant for diagnostic to establish the appropriate matching control group. Otherwise, the accuracy of the diagnostic test could be overestimated. However, as we all know, the meta-analysis dependent on the primary studies. Base on the current study status, the only thing we can do is point out the direction for the future research. Although the non-restrict design could overestimate the discrimination power of the s-p53-antibody in EC, the meta-analysis which base on comprehensive, large sample quantitative assessment can provide more convincing evidence. Indeed, the evidence is compelling in that s-p53 antibody assay specificity were higher than 0.9 in all of the 15 included studies, ranging from 0.91 to 1.00. Third, although we did not observe significant publication bias between studies, it is uncertain whether some data were missed because of unpublished studies. Missing information may report lower diagnostic of s-p53-antibody.

Our study represents a new trend in diagnosis of the cancer: convenient, noninvasive, low costs biomarkers will play a significant role in screening cancer. Future studies should focus on the following tasks: (i) improve the sensitivity and specificity of the detection method, (ii) use blood, serum or other convenient samples, (iii) standardize the detection method and cut-off, and (iv) conduct normative diagnostic tests or collect samples from cases before biopsies or at least before treatment to improve sensitivity. These tasks will reduce the heterogeneity among studies, enabling us to conduct an accurate meta-analysis to find the diagnostic value of the s-p53 antibody. Furthermore, more studies are greatly needed to examine the association between s-p53 antibody and the stage and the prognosis of the EC. This will help avoid the unnecessary treatment, as EC therapies are associated with significant adverse effects that impact patient health and quality of life.

In conclusion, the current evidence suggests that s-p53 antibody has potential diagnostic value though currently provides low sensitivity. Patients with esophageal cancer have higher chance of being s-p53 antibody test-positive compared with patients without EC. We believe that s-p53-antibody may be useful for monitoring residual tumor cells and for aiding in the selection of candidates for less invasive treatment procedures because of the high specificity of s-p53-antibody. Further studies may need to identify patterns of multiple biomarkers to further increase the power of EC detection.

## Supporting Information

Figure S1 **Methodological quality graph: review of authors' judgments about each methodological quality item presented as percentages across all 15 included studies.**
(TIF)

Figure S2 **Forest plot of sensitivity and specificity of 15 individual studies for s-p53-antibody in the diagnosis of EC.** The point estimates of sensitivity from each study are shown as solid circles. Error bars are 95% confidence intervals.
(TIF)

Figure S3 **Forest plot of estimates of positive likelihood ratio (PLR) and negative likelihood ratio (NLR) for s-p53-antibody in the diagnosis of EC.** The point estimates of positive likelihood ratio from each study are shown as solid circles. Error bars are 95% confidence intervals.
(TIF)

Table S1 **Search strategy in PubMed. Footnote: ESCC: esophageal squamous cell carcinoma; EAC: esophageal adenocarcinomas cancer; OSCC: oesophageal squamous cell carcinoma; OAC: oesophageal adenocarcinomas cancer.** Search time limits: May 31st, 2012.
(DOC)
