# Supplementary figures and images for: Potential Diagnostic Value of Serum p53 Antibody for Detecting Esophageal Cancer: A Meta-Analysis
Source: PLoS One. 2012 Dec 28;7(12):e52896. doi: 10.1371/journal.pone.0052896 (PMC3532438; doi:10.1371/journal.pone.0052896)

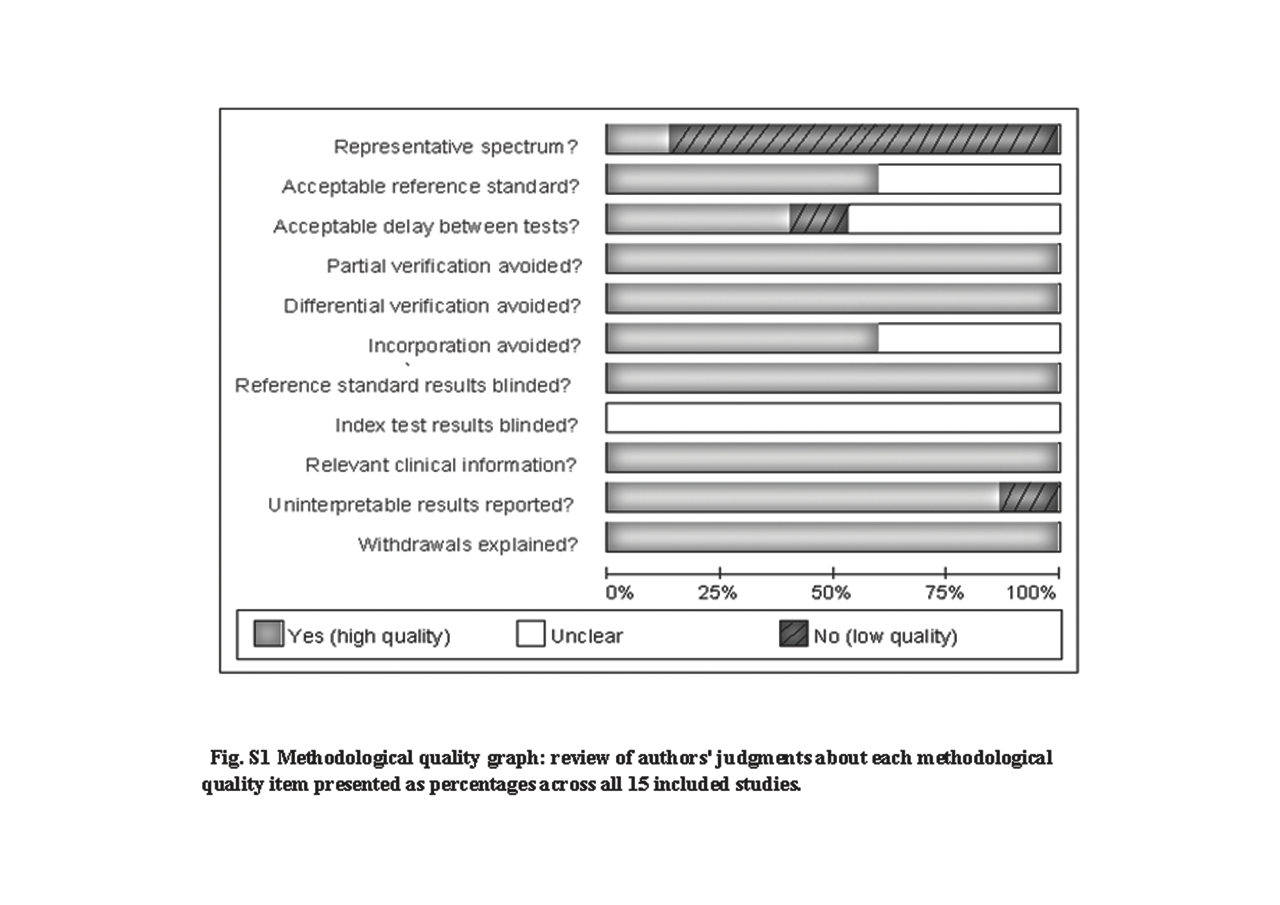

Supplement: Figure S1 — Methodological quality graph: review of authors' judgments about each methodological quality item presented as percentages across all 15 included studies. (TIF) [file pone.0052896.s001.tif]

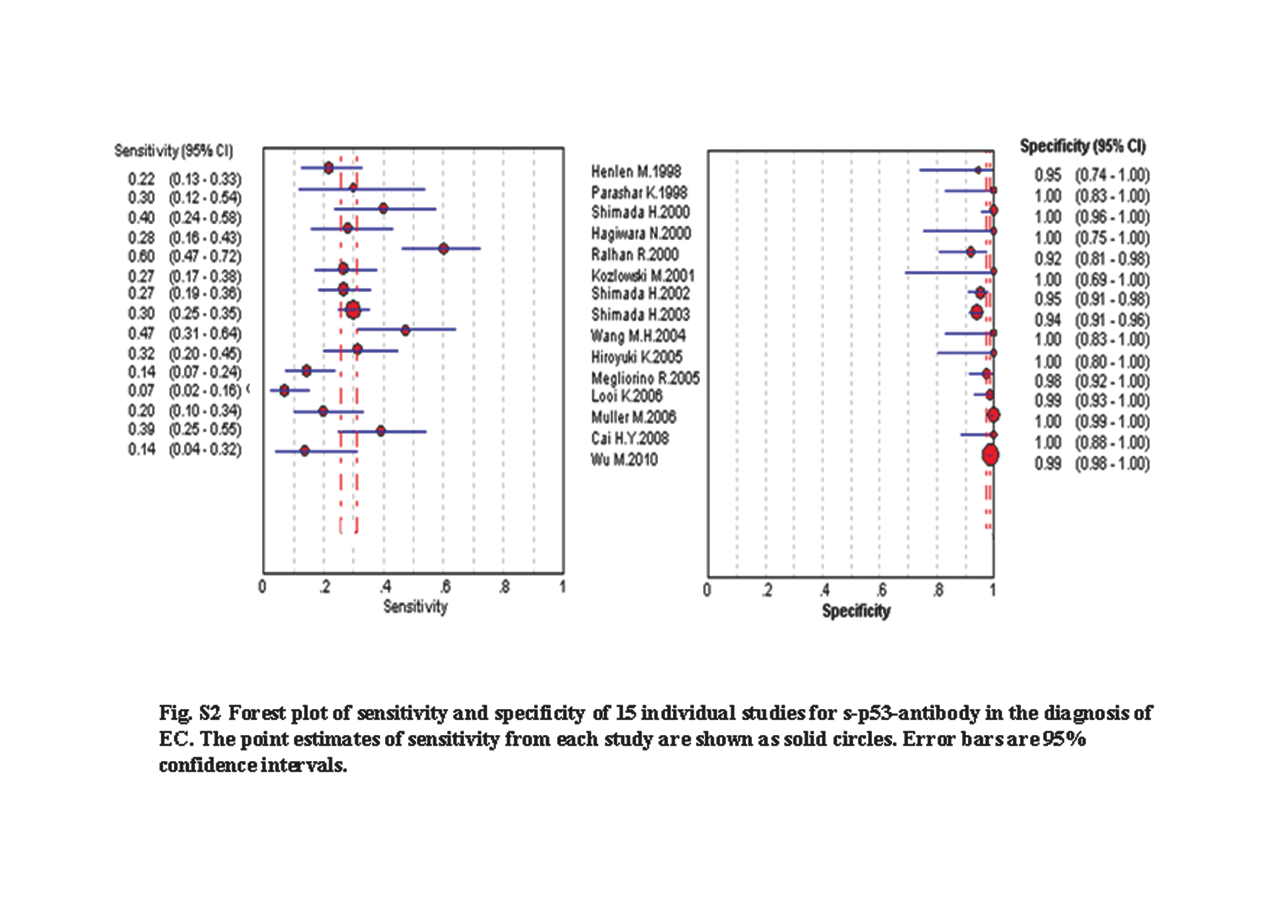

Supplement: Figure S2 — Forest plot of sensitivity and specificity of 15 individual studies for s-p53-antibody in the diagnosis of EC. The point estimates of sensitivity from each study are shown as solid circles. Error bars are 95% confidence intervals. (TIF) [file pone.0052896.s002.tif]

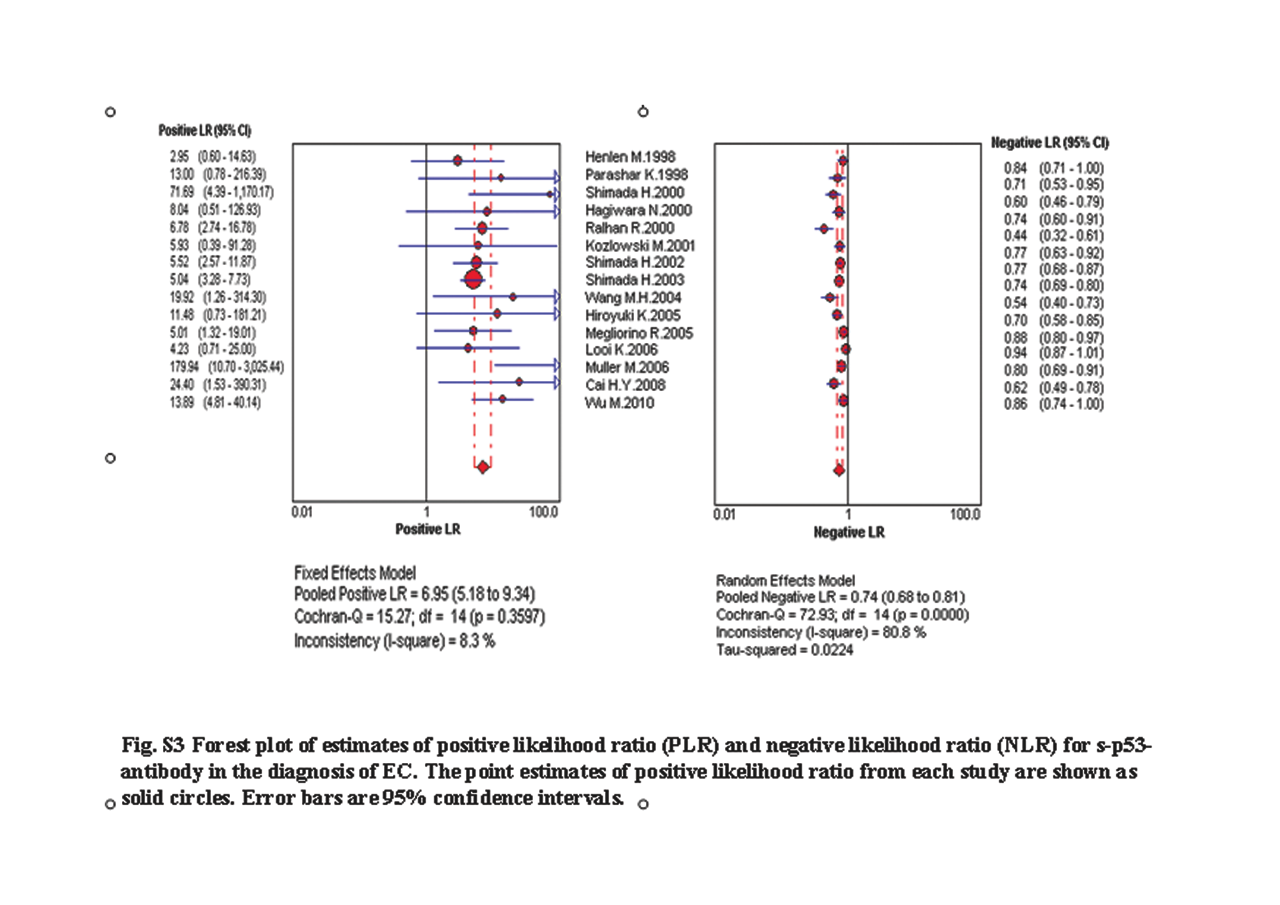

Supplement: Figure S3 — Forest plot of estimates of positive likelihood ratio (PLR) and negative likelihood ratio (NLR) for s-p53-antibody in the diagnosis of EC. The point estimates of positive likelihood ratio from each study are shown as solid circles. Error bars are 95% confidence intervals. (TIF) [file pone.0052896.s003.tif]
