# Supplementary material for: Potential Diagnostic Value of Serum p53 Antibody for Detecting Esophageal Cancer: A Meta-Analysis
Source: PLoS One. 2012 Dec 28;7(12):e52896. doi: 10.1371/journal.pone.0052896 (PMC3532438; doi:10.1371/journal.pone.0052896)
Supplement: Table S1 — Search strategy in PubMed. Footnote: ESCC: esophageal squamous cell carcinoma; EAC: esophageal adenocarcinomas cancer; OSCC: oesophageal squamous cell carcinoma; OAC: oesophageal adenocarcinomas cancer. Search time limits: May 31st, 2012. (DOC) [file pone.0052896.s004.doc]

## Table S1 Search strategy in PubMed

| Search | Query | Items found |
| --- | --- | --- |
| #1 | esophageal neoplasms[all fields] | 39789 |
| #2 | esophageal or esophagus or oesophagus or oesophageal[all fields] | 134738 |
| #3 | cancer or carcinoma or adenocarcinoma malignan or tumor or tumour | 2915713 |
|  | or neoplasm[all fields] |  |
| #4 | #2 and #3 | 52160 |
| #5 | **ESCC OR EAC OR OSCC OR OAC** [all fields] | 8129 |
| #6 | **barrett esophagus** | 5764 |
| #7 | #1 or #4 or #5 or #6 | 60179 |
| #8 | blood OR serum OR serological OR seropositive OR seropositivity | 3877732 |
|  | OR serum antibody OR sera OR plasma[all fields] |  |
| #9 | P53 or TP53 | 65211 |
| #10 | #7 and #8 and #9 limits:humans | 103 |

Note: ESCC: esophageal squamous cell carcinoma; EAC: esophageal adenocarcinomas cancer; OSCC: [oesophageal](app:ds:oesophageal) squamous cell carcinoma; OAC: [oesophageal](app:ds:oesophageal) adenocarcinomas cancer.

# Search time limits : May 31st, 2012
